# C-terminus Methionene Specifically Involved in Binding Corn Odorants to Odorant Binding Protein4 in *Macrocentrus cingulum*

**DOI:** 10.3389/fphys.2017.00062

**Published:** 2017-02-08

**Authors:** Tofael Ahmed, Tiantao Zhang, Zhenying Wang, Kanglai He, Shuxiong Bai

**Affiliations:** ^1^State Key Laboratory for the Biology of the Plant Diseases and Insect Pests, Institute of Plant Protection, Chinese Academy of Agricultural SciencesBeijing, China; ^2^Bangladesh Sugarcrop Research InstitutePabna, Bangladesh

**Keywords:** *M. cingulum*, OBPs, expression patterns, mutagenesis, odorant-protein binding assay

## Abstract

The soluble carrier proteins, OBPs carry odor components through sensilium lymph to specific receptors within the antennal sensilla to trigger behavioral responses. Herein, McinOBP4 was characterized from the *Macrocentrus cingulum*, which is the specialist parasitic insect of *Ostrinia furnacalis* for better understanding of olfactory recognition mechanism of this wasp. The classical odorant binding protein McinOBP4 showed good binding affinity to corn green leaf volatiles. RT-qPCR results showed that the McinOBP4 was primarily expressed in male and female wasp antennae, with transcripts levels differing by sex. Fluorescence assays indicate that, McinOBP4 binds corn green leaf volatiles including terpenoides and aliphatic alcohols as well as aldehydes with good affinity. We have also conducted series of binding assay with first mutant (M1), which lacked the last 8 residues and a second mutant (M2), with Met119 replaced by Leucine (Leu119). In the acidic conditions, affinity N-phenylnaphthylamine (1-NPN) to McinOBP4 and M1 were substantially decreased, but increase in basic condition with no significant differences. The lack of C-terminus showed reduced affinity to terpenoides and aliphatic alcohols as well as aldehydes compounds of corn odorants. The mutant M2 with Met119 showed significant reduction in binding affinity to tested odorants, it indicating that Met119 forming hydrophobic chain with the odorants functional group to binding. This finding provides detailed insight of chemosensory function of McinOBP4 in *M. cingulum* and help to develop low release agents that attract of this wasp to improve ecologically-friendly pest management strategy.

## Introduction

The sense of smell is important for insects to detect chemicals cues in their surrounding environment and controlling fundamental behaviors as foraging, host-seeking, mating choice, oviposition site locating by females, warning, and defense as well as avoiding threats. The chemo smells from the green leaf volatiles, pheromones, and host/predator odors captured by chemoreceptors and convert into physiological signals that changing insect behaviors (Jacquin-Joly and Merlin, 2004). The basis of the observed sensitivity and selectivity of insects to particular odor has been studied by researchers who attempt to develop environmentally friendly pest management strategies which could be alternative to chemical based controlling. Membrane proteins such as odorant receptor (ORs), ionotropic receptors (IRs), and sensory neuron membrane proteins (SNMPs) play central roles in translating the chemo signals to an electrical signal, while odorant binding proteins (OBPs), or chemosensory proteins (CSPs) are proposed to bound, convey, and even recognize exact pheromones and odorants to their specific receptors (Jacquin-Joly and Merlin, 2004; Leal, 2013; Sun et al., 2016).

In the 1980s, OBP was discovered in the giant moth *Antheraea polyphemus* (Vogt and Riddiford, 1981). More than 30 years since their discovery, many studies related to structure and function have been undertaken (Vogt and Riddiford, 1981; Vogt et al., 2002; Pelosi, 2005; Pelosi et al., 2006), however, the mode of action and specific role of OBPs in the chemoreception were still not clear (Sun et al., 2013; Ahmed et al., 2014). The ligand binding mechanisms have been explored through key amino acid residues, three dimensional structures and their physiochemical properties (Zhou et al., 2009; Lagarde et al., 2011). The 3D structure of the *Bombyx mori* PBP (BmorPBP) complexed with bombykol, was the first to be studied. The helices of α1, α4, α5, and α6, forms a binding cavity and the C-terminus promote a α-helix at pHs <6.0 and folds back into the cavity, but this α-helix withdraws from the binding cavity and convert in available form for the pheromones at pH 7.0 (Damberger et al., 2000; Sandler et al., 2000; Horst et al., 2001). The C-terminus in ApolPBP1, forming the seventh α -helix, played crucial role in odorant binding and/or locking the odor molecules in the binding cavity as well (Katre et al., 2013). Previous studies reported this type of conformational changed in several OBPs (Damberger et al., 2007; Sun et al., 2013). The short length of their C-terminus OBPs, cannot make an additional helix that extends into the binding cavity, but covers a portion, and may contribute to the binding of specific ligands (Tegoni et al., 2004; Leite et al., 2009). CquiOBP1 and OBP that lacks a C-terminus binds to the oviposition pheromone, MOP, using a pH-dependent binding/releasing model. CquiOBP1 makes a small central cavity for the lactone head group and a long hydrophobic channel for the C-terminus (Mao et al., 2010). So, OBPs were suggested to probably exhibit different ligand binding and releasing mechanisms.

The most critical amino acid residues have been identified for binding ligands to different insect OBPs (Sandler et al., 2000; Jiang et al., 2009; Zhou et al., 2009; Sun et al., 2013; Ahmed et al., 2014; Zhuang et al., 2014; Yin et al., 2015). *Drosophila* LUSH binds the specific ligand vaccenyl acetate, in which a single amino acid residue is involved when this protein undergoes a conformational change. Such type of change was sufficient to triggers binding of ligand to the specific OR (Sun et al., 2013), and generate an electrophysiological response by the OR67d in the T1 neuron (Laughlin et al., 2008). The LUSH mutant clearly showed that kind of mechanism, which enhance the protein conformation when binding to cVA (Laughlin et al., 2008). The C-terminus Lys123 of *Helicoverpa armigera* OBP7, HarmOBP7 and Glu130 of *Macrocentrus cingulum* OBP1, McinOBP1 form hydrogen bonds with the functional groups of their odorants and are probably involved in binding these compounds (Ahmed et al., 2014).

The specialist larval polyembryonic endoparasitoid of *M. cingulum* Brischke (Hymenoptera: Braconidae) is considered for the genus *Ostrinia* (Lepidoptera: Crambidae) including the ACB, *O. furnacalis* (Guenée) (Lepidoptera: Crambidae) and ECB, *O. nubilalis* (Hübner) (Edwards and Hopper, 1999; Hu et al., 2003; Ahmed et al., 2013), which are the most destructive insect pests of corn (*Zea mays* L.) in the world (Bourguet et al., 2014; Wang et al., 2014). Several factors such as temperature, humidity, geographical location and pathogen infection rate are considered to influence the parasitic efficacy of *M. cingulum*. Parasitization efficacy range to 80% in the in Northern France but it depends on several conditions (Hartlieb and Anderson, 1999; Xu et al., 2010). The OBPs play crucial roles in insect olfactory systems, till functional characterization of only one OBP has been done for *M. cingulum* (Ahmed et al., 2014). From the fluorescence binding assays with different corn odorants, the McinOBP1 had strong binding ability to corn odorants that suggested McinOBPs may play important roles in this wasp olfaction system (Ahmed et al., 2014). However, except McinOBP1, functional study of *M. cingulum* olfactory related protein remains uninvestigated. In our present research on McinOBP4, a novel classical OBP and findings of transcript level on different tissues, site directed mutagenesis, and binding affinity to corn odorants that are composed of aldehydes, terpenoids, and aliphatic alcohols along with others aromatic compounds would provide functional mechanisms in details.

## Materials and methods

### Sample collection and RNA extraction

The parasitoid wasp, *M. cingulum* Brischke were reared at 25°C, in 16 h light:8 h dark on the host insect, *O. furnacalis* in the laboratory. Adult parasitoid wasps were fed with 20% honey solution. The antennae of 3 days-old male and female (200 of each sex) adults were excised at the base and immediately transferred into tubes immersed in liquid nitrogen. The preparation of all other samples including the head (without antennae), thorax, leg, and abdomen were carried out in the same way. Prepared samples were stored at −80°C until used. The total RNA of each sample was isolated according to the manual of TRIzol reagent (Invitrogen, Carlsbad, CA, USA). The RNA integrity was verified by 1% agarose gel electrophoresis and quantity was assessed with a Nanodrop ND-2000 spectrophotometer (NanoDrop products, Wilmington, DE, USA). First-strand cDNAs were synthetized by reference to the handbook of EasyScript First-Strand cDNA Synthesis SuperMix (TransGen Biotech, Beijing, China), and employed as templates for latter PCR amplification.

### McinOBP4 sequence analysis

The McinOBP4 has been identified from the antennal transcriptome analysis (GenBank no. KY270854). The SignalP 3.0 program (http://www.cbs.dtu.dk/services/SignalP/) was used to predicted signal peptides from N-terminal. The McinOBP4 and other insects OBPs amino acids sequences were align with ClustalX 1.83. MEGA 5.2 software was used to construct phylogenetic tree followed by the neighbor-joining method (Tamura et al., 2007).

### Polymerase chain reaction (PCR)

One μL cDNA with gene specific primers were amplified in a TC-5000 thermal cycler (Bibby Scientific Ltd, UK) as follows:

McinOBP4-sense: 5′- CGGATCCAAGCGACCAGATTTC -3′ and
McinOBP4-antisense: 5′- CGAAGCTTTTACACCATGAACCAC -3′

The *BamH*I and *Hind*III are employed in the sense and antisense primers, respectively. The PCR amplification conditions are 3 min at 94°C for denaturation, 30 s at 94°C, 30 s at 57°C and 1 min at 72°C for 37 cycles and last 10 min at 72°C.

### McinOBP4 gene cloning

The pGEM-T easy vector was used into PCR gel extraction products ligation and kept at 4°C for overnight. The ligation products were transformed into Trans1-T1 competent cell (TransGen Biotech, Beijing, China) which belonged to *E. coli*. Positive colonies were selected by PCR using with M13 (F and R) primers, and cultured in ampicillin enriched LB liquid medium. The custom sequencing was at Sangon Biotech, Beijing, China.

### Expression of McinOBP4 and purification

The McinOBP4 sequence with endonuclease restriction sites *Bam*HI and *Hind*III were incorporated into PET-32a(+), and the generated constructs were transformed into BL21 (DE3) *E. coli* strains and cultured on ampicillin enriched solid LB medium. Positive clone was grown in medium with ampicillin at 220 rpm in 37°C for 8 h.

Recombinant McinOBP4 protein was induced with 0.5 mM Isopropyl β-D-1-thiogalactopyranoside (IPTG) under the condition of 37°C, 160 rpm, and purification of the recombinant McinOBP4 was initiated by harvesting of McinOBP4-expressing DE3 cells from 1 liter cultures by centrifugation. All expressed proteins were found in the supernatant, and the recombinant proteins were purified by Ni ion affinity chromatography (GE-Healthcare, Sangon Biotech Co., Ltd., Shanghai, China). SDS-PAGE was used to monitor protein expression and purification. The purified proteins were stored at −20°C in 50 mM Tris-HCl buffer with a pH of 7.4.

### Preparation of mutants

The C-terminal tail truncated form of the McinOBP4 was obtained by removing the last 8 amino acids and inserting a stop codon after Leu112. The McinOBP4-Wt/pGEM-T Easy construct was used as a template. A pair of primer was designed manually and as listed sense primer 5′- CGGATCCAAGCGACCAGATTTC -3′ (underlined with *Bam*HI) and antisense primer, 5′- CGAAGCTTTTACTTCGATTGCAC -3′ (underlined with *Hind*III) where 113th position residues used as a stop codon. The mutant M2 (Met119) was prepared along with the pair of primer as sense primer 5′- CGGATCCAAGCGACCAGATTTC -3′ (under lined with *Bam*HI), and antisense primere 5′- CGAAGCTTTTACACCAAGAACCACATC -3′ (under lined with *Hind*III). The following reaction conditions were employed: 4 min at 94°C for initial denaturation, followed by 36 cycles of 30 s at 94°C, 30 s at 57°C and 1 min at 72°C for 36 cycles and 10 min at 72°C. The selected mutant was cloning, expression; protein induction and purification were done according to our previous works (Ahmed et al., 2014).

### Distribution pattern of McinOBP4

The qRT-PCR was employed to measure the tissue-specific transcripts level of McinOBP4 by using cDNA from antennae, head, leg and abdomen from female and male wasps. The gene specific primers, F: 5′-GCGACCAGATTTCGTGACT-3′; R: 5′-TGTCGTTTGGAATATCGCC-3′ were used. The ABI 7500 fast real-time PCR system (Applied Biosystems, USA) were employed according to our previous study (Ahmed et al., 2014).

### Fluorescence measurements

One cm light path fluorimeter quartz cuvette was used on a FluoroMax-4 (Horiba Scientific, USA) spectrophotometer at room temperature for fluorescence spectra recording and excitation wavelength of 337 nm was chosen and 300–500 nm for the emission with a 5 nm slit width. Spectra were recorded with 2 μM McinOBP4 in 50 mM Tris-HCl of different pH value, while ligands were added as 1 mM methanol solutions.

### Competitive binding assay

To measure the affinity of the fluorescence ligand N-phenyl-1-naphthylamine (1-NPN) to McinOBP4-wt and mutants, a 2 μM solution of the protein in 50 mM Tris-HCl, pH 7.4, was titrated with aliquots of 1 mM ligand in high-performance liquid chromatography (HPLC) purity grade methanol to final concentrations of 2–20 μM. The probe was excited at 337 nm and emission spectra were recorded between 300 and 500 nm. While the other competitor were measured in competitive binding assays, where 1-NPN using as the fluorescent reporter at 2 μM concentration and each competitor (odorants) over concentration ranges 2–14 μM, depending on the different odorants.

### Dissociation constants (K_D_) calculation

It was assumed that McinOBP4 was 100% active and the protein-ligand binding was 1:1 ratio at saturation. The obtained data were processed with Graphpad Prism software (Graphpad Software, Inc.) and using a software package SPSS Version 16 (http://www.spss.com). The maximum intensity values of emission were plotted against the 1-NPN concentration for the K_D_ determination, and the values of intensity were used for the evaluation the bound ligands. The curve was linearized using Scatchard Plots to verify the confidence of K_D_. IC_50_ for other corn odorants were calculated using with Microsoft excel 2010, and the equation, *K*_D_ = [IC_50_]/[1+(1–NPN)/K_1−NPN_] was used for the *K*_D_ values calculation. In this equation [IC_50_] stands for the ligand concentration where the ligand quenching the fluorescence intensity of 1-NPN to 50%, [1-NPN] and K_1−NPN_ mean free 1-NPN concentration and McinOBP4/1-NPN complex *K*_D_ values, respectively (Campanacci et al., 2001; Ban et al., 2002).

### Molecular modeling and docking

Three dimensional model of McinOBP4 was generated through the I-TASSER web server (http://zhanglab.ccmb.med.umich.edu/I-TASSER/.) (Wu et al., 2003; Roy et al., 2010). The protein sequences on RCSB Protein Data Bank (PDB) (http://www.rcsb.org) were employed to compare the McinOBP4 sequence. The AmelPBP1 (Leite et al., 2009) model was used as template for the structure of McinOBP4. Molecular dynamics program CDOCKER (Wu et al., 2003) was used to investigate the binding characteristics of two corn odorants, limonene, and trans-3-hexen-1-ol-acetate at structural levels and total interaction energy was calculated from these two odorants and respective residues of McinOBP4.

## Results

### Characteristics of McinOBP4 protein

**Figure 1:**
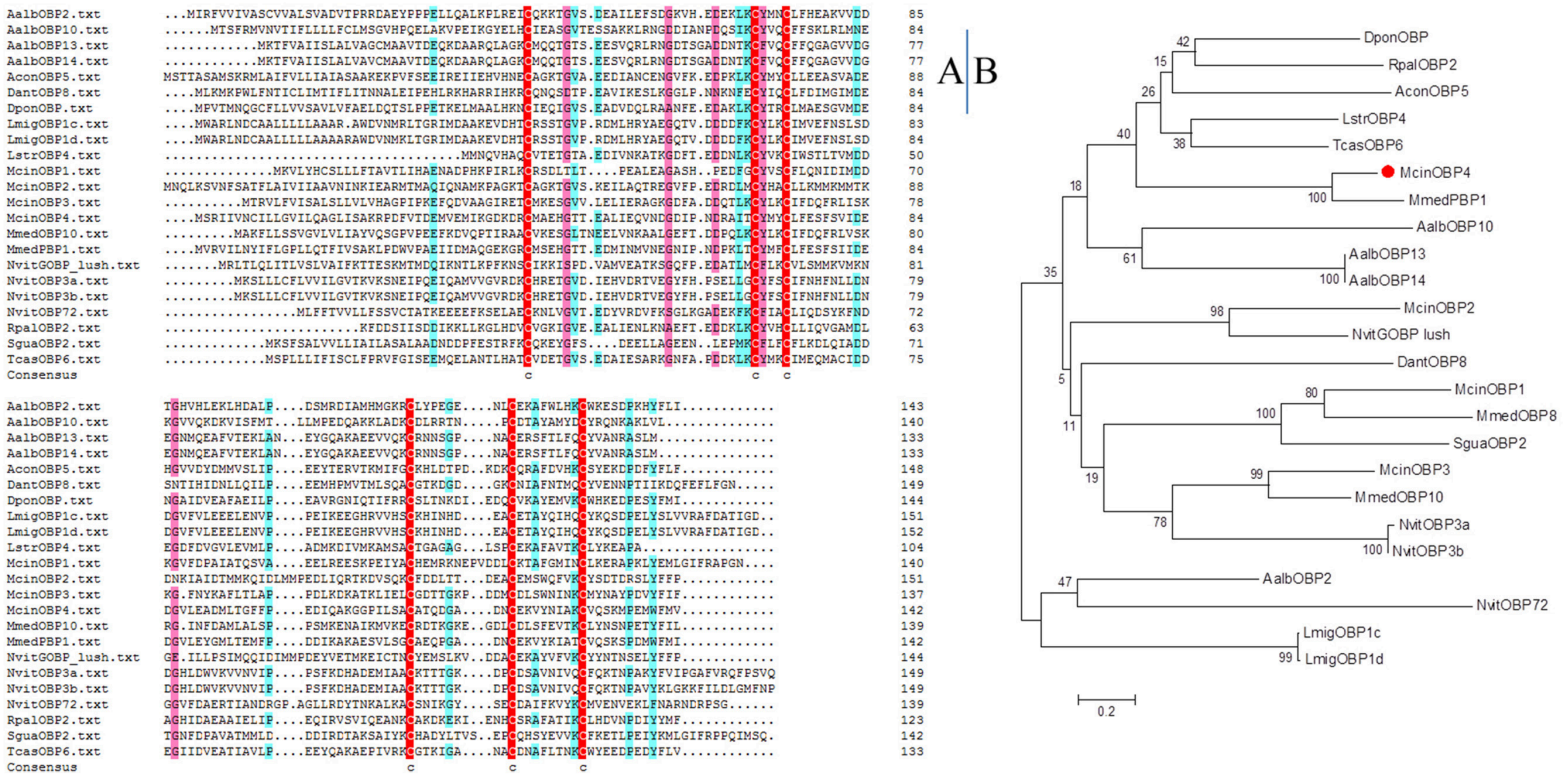
**Sequence alignments and Phylogenetic analysis of McinOBP4. (A)** Alignment of McinOBP4 from hymenopteran insects as well as other insects. **(B)** Neighbor-joining phylogenetic tree was constructed used MEGA 6.0 with a p-distance model and a pairwise deletion of gaps.

McinOBP4 was identified in the antennal transcriptomic analysis, is a protein highly expressed in olfactory tissues specifically expressed in antennae. Through the biochemical approach full length McinOBP4 was cloned. McinOBP4 encoded 143 amino acids residues including a 22-aa-long signal peptide, with a predicted MW of 15.71 kD. The theoretical isoelectric points and molecular weight of the mature proteins were 4.00 and 13.43 kD, respectively. The amino acid sequences alignment of McinOBP4 with the Hymenopteran and other insects (Figure 1A). To analyze the phylogenetic relationship of McinOBP4 with other insects, we constructed phylogenetic tree comprising 24 OBPs from different insect species (Figure 1B). McinOBP4 was phylogenetically closest to MmedPBP1 from *Microplitis mediator*, which belongs to the same order. This result suggested that McinOBP4 is a classical OBP/PBP, need to further study to confirm.

### Tissue specific expression of McinOBP4

**Figure 2:**
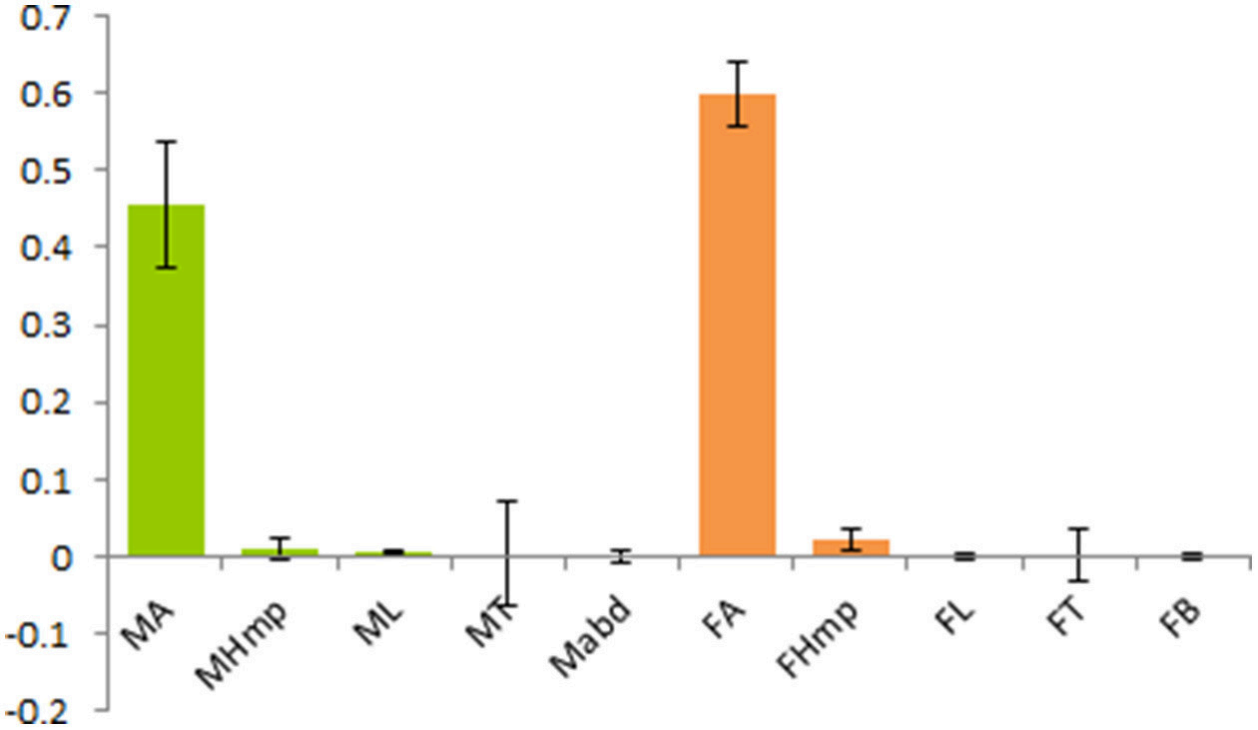
**Relative transcript levels of McinOBP4 of both sexes**. Expression level of *M. cingulum* odorant binding protein 4 (McinOBP4) in different tissues of adult male and females measured by qPCR. cDNAs were amplified with specific primers from antennae, heads (without antennae), thoraxes, abdomens, legs, and abdomen.

Real-time quantitative PCR (RT-qPCR) was employed to check the transcripts level of McinOBP4 in male and female antennae, de-antennated heads, legs, thoraxes, and abdomens. Tissue specific tRNA were isolated from male and female wasps, and the reverse transcribed. The RT-qPCR results showed that the target protein was largely expressed in antennae of both sex, with low in other tissues, might be the McinOBP4 was specifically expressed in adult antennae (Figure 2). Generally, very low transcripts levels were in other test tissues except for the adult antennae.

### Odorant binding of recombinant McinOBP4-Wt

**Figure 3:**
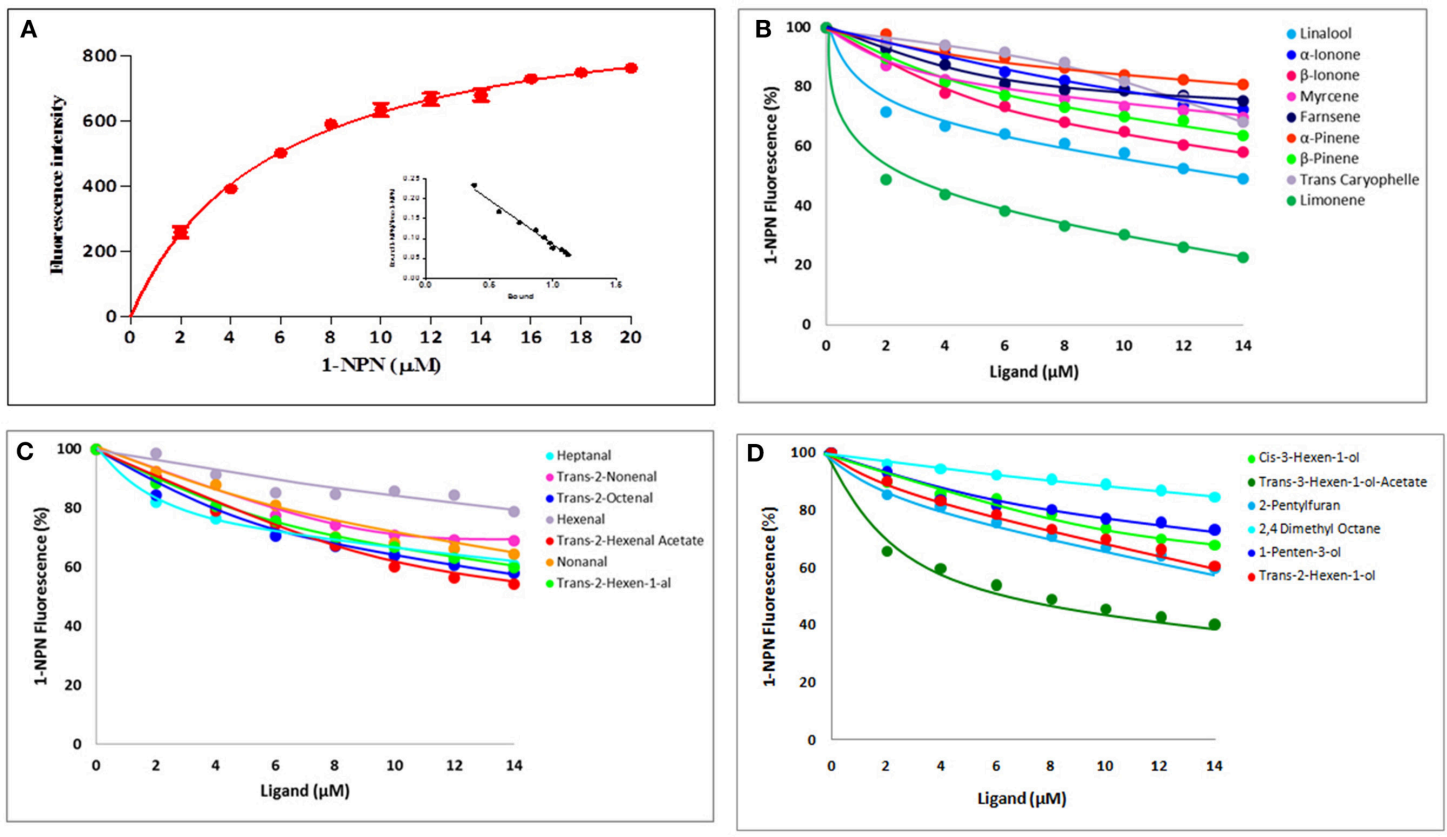
**Competitive Binding of 1-NPN and corn odorants to McinOBP4. (A)** Binding curves and relative Scatchard plot (insert) of 1-NPN and McinOBP4 protein. **(B)** Competitive binding affinities of McinOBP4-Wt to different terpenoids corn odorants. **(C)** Binding affinities of various aldehyde odorants to McinOBP4-Wt and **(D)** Affinities of McinOBP4-Wt to aliphatic alcohol and others.

We measured affinities of 1-NPN with purified McinOBP4. According to the changes in fluorescence intensity, McinOBP4 bound strongly to 1-NPN with binding affinity of 5.69 ± 0.83a μM (Figure 3A). The binding affinities of McinOBP4-Wt to corn odorants were tested belongs the three groups. List of odorants and resulted affinities curves are shown in Table 1 and Figures 3B–D, respectively. The results suggested that most of the selected corn odorants displaced 1-NPN from the McinOBP4-Wt/1-NPN complex. Among the tested compounds, limonene and trans-3-hexen-1-ol-acetate had the highest affinities to McinOBP4-Wt with *K*_D_ values of 4.12 ± 0.70 and 6.99 ± 1.23 μM respectively (Figures 3B,D). The linalool, trans-2-octanal, and β-ionone had medium affinities to McinOBP4-wt, with K_D_ of 9.61 ± 0.23, 11.78 ± 0.57, and 11.92 ± 0.49 μM, respectively.

**Table 1:** **Binding affinities of corn odorants to the recombinant McinOBP4-Wt proteins at ***pH*** = 7.4**.

**Corn Odorants**	**CAS Number**	**IC_50_**	**K_D_**
**ALDEHYDES**			
Heptanal	111-71-7	16.75	13.03
Trans-2-Nonenal	18829-56-6	16.46	12.81
Hexenal	66-25-1	34.58	26.91
Nonanal	124-19-6	16.07	12.51
Trans-2-Hexenal Acetate	6728-26-3	17.04	13.26
Trans-2-Hexen-1-al	6728-26-3	16.43	12.79
Trans-2-Octenal	2548-87-0	15.33	11.93
**TERPENOIDS**			
Linalool	78-70-6	12.35	9.61
α-Ionone	127-41-3	24.09	18.75
β-Ionone	14901-07-6	15.14	11.78
Myrcene	123-35-3	22.98	17.89
Farnsene	502-61-4	27.40	21.31
α-Pinene	80-56-8	34.18	26.60
β-Pinene	19902-08-0	18.85	14.67
Trans Caryophelle	87-44-5	24.49	19.06
Limonene	138-86-3	5.29	4.12
**ALIPHATIC ALCOHOLS AND OTHERS**			
Cis-3-Hexen-1-ol	3681-71-8	19.88	15.47
Trans-3-Hexen-1-ol-Acetate	928-97-2	8.98	6.99
2-Pentylfuran	3777-69-3	20.12	15.66
2,4 Dimethyl Octane	4032- 94-4	47.00	36.57
1-Penten-3-ol	616-25-1	25.39	19.76
Trans-2-Hexen-1-ol	3681-71-8	19.83	15.43

**Figure 4:**
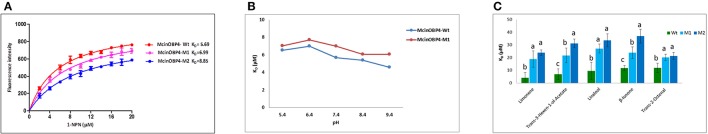
**Binding Affinities of 1-NPN and corn odorants to McinOBP4 mutants. (A)** Affinities of 1-NPN to the McinOBP4-Wt and two mutants. Dissociation constants (K_D_) were 5.69 ± 0.83 μM for McinOBP4-Wt, 6.99 ± 0.59 μM for McinOBP4-M1, 8.85 ± 0.63 μM for McinOBP4-M2 are shown in inset. **(B)** The dissociation constants of the complexes with 1-NPN of McinOBP4-Wt and C-truncated mutant McinOBP4-M1 at different pH level. At neutral basic pH level, both proteins showed a maximum activity. **(C)** Dissociation constants of complexes between McinOBP4-Wt and its two mutants (McinOBP4-M1 and M2) and the five selected odorants. These two mutants showed lower binding affinity to selected corn odorants with respect to the wild type, indicating that the respective amino acid is involved in binding these corn odorants.

Additionally, we investigated the affinity of 1-NPN to McinOBP4-Wt and McinOBP-M1 over the pH range from 4.4 to 9.4. Binding curves are showed in Figures 3A–D at pH 7.4 and the wild and mutant proteins affinities at several pH levels (Figure 4B). We did not observe significant loss of binding activity in acidic condition (pH 4.4) while a basic condition increase in affinity was measured. At different pH levels, the C-terminal truncated protein has no effect of their activities. Moreover, we have measured binding affinity of the corn odorant to the mutant McinOBP4-M1 (Figure 4C). The corn odorants, showed poor binding abilities to the M1 mutant compared to the McinOBP4-Wt, the results unlike to 1-NPN, which affinity was not depends on the segments of C-terminus.

### Mutant binding assay

We prepared a second mutant to identify one of key amino acid residue by mutated Met119 to leucine, predicted candidates forming a Schiff base with the aldehyde group or hydrogen bonds with other odorants compounds. We reasoned that Phe118 could establish an electrostatic interaction with Met116, and Met113 could be linked to Glu115. This would leave Met119 free to interact with the odorants. Furthermore, limonene and trans-3-hexen-1-ol-acetate were bound in a large flask-shaped hydrophobic pocket and the Met13, Gly38, Lue53, Val59, Glu62, Val67, Leu71, Phe74, Ile79, Gly83, Leu87, Glu93, and Met119 residues were take part in binding to these corn odorants. Residues and odorants interaction energy are shown in Table 2. By the total interaction energy comparison, limonene was showed a more interaction energy than trans-3-hexen-1-ol-acetate, especially at Met119 (Table 2). This phenomenon leads higher binding affinity (lower K_D_) of McinOBP4-limonene complex. Additionally, validated key binding sites through the sequence alignment of McinOBP4 to other insects OBPs such as Val14 of LstiGOBP1 (Yin et al., 2015), IIe80 to HoblOBP1 (Zhuang et al., 2014) and V87A to LmigOBP1 (Jiang et al., 2009; alignment results not shown in here).

**Table 2:** **Total interaction energies (kcal/mol) from McinOBP4 key residues and selected corn odorants**.

**Residues**	**Limonene**			**Trans-3-Hexen-1-ol-Acetate**		
	***E*****_total_**	***E*****_vdw_**	***E*****_ele_**	***E*****_total_**	***E*****_vdw_**	***E*****_ele_**
Met13	−0.763	−0.292	−0.471	−0.916	−0.913	−0.003
Gly38	−1.472	−1.345	−0.127	−2.197	−1.351	−0.846
Lue53	−1.077	−0.979	−0.098	–	–	–
Val59	−1.1149	−1.0369	−0.078	−1.5	−1.039	−0.461
Glu62	–	–	–	−0.23	−0.203	−0.027
Val67	−0.932	−0.929	−0.003	−1.068	−0.155	−0.913
Leu71	−0.924	−1.133	0.209	–	–	–
Phe74	−1.504	−1.412	−0.092	−0.956	−0.951	−0.005
Ile79	−0.676	−0.495	−0.181	−0.652	−0.615	−0.037
Gly83	−0.887	−0.991	0.104	−1.496	−0.881	−0.615
Leu87	−0.245	−0.292	0.047	–	–	–
Glu93	−1.214	−1.307	0.093	−1.136	−1.219	0.083
Met119	−2.797	−2.351	−0.446	−2.939	−1.993	−0.946

*E_total_, total interaction energy; E_vdw_, Van der Waals energy; E_ele_, electrostatic interaction energy*.

The same fluorescence probe 1-NPN was used under the previously described conditions. The M2 bound 1-NPN with a K_D_ of 8.85a μM, little more than the McinOBP4-Wt; it indicated that McinOBP4-M2 and 1-NPN interaction was not influenced by the mutation (Figure 4A). Binding affinities of the mutants M2 was then evaluated with five corn odorants namely limonene and trans-3-hexen-1-ol-acetate, linalool, trans-2-octanal, and β-ionone and are shown in Figure 4C. Compared to wild type and first mutant, the second mutant showed lower binding affinities to the tested odorants.

### Molecular modeling

**Figure 5:**
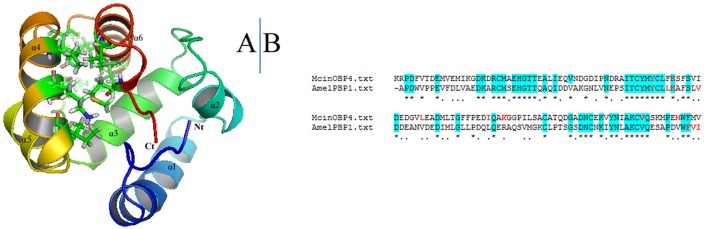
**3D model structure of the McinOBP4. (A)** 3D structure of McinOBP4 was built on the reference of structure of PBP1 from the Honey bee *Apis mellifera*. Six α-helixes, N-terminal (Nt), and C-terminal (Ct) are marked. **(B)** Sequence alignment of McinOBP4 and AmelPBP1.

McinOBP4 sequence was compared to all known proteins sequences in the protein data bank through a Blastp search; the AmeliBP1 as template 3D structure to the McinOBP4 in I-TASSER. The best model was chosen from 10 candidates through homology modeling. The verification score and Ramachardran plot were used for assessed the quality of the best model (data not shown). McinOBP4 model C-score were 0.83 (ranges −5 to 2) suggests a high level of confidence in the predicted model (Roy et al., 2010). Estimated TM-score and RMSD of predicted model McinOBP4 are 0.83 ± 0.08 and 2.7 ± 2.0 A°, respectively. The 3D structure of McinOBP4 (Figure 5) was composed with six α-helices which are located into the 8–24 (α1), 29–36 (α2), 46–57 (α3), 68–74 (α4), 77–89 (α5), and 98–111 (α6) residues. Four (α1, α2, α3, α4, and α5) antiparallel helices were converged to form a large flask-shaped hydrophobic binding cavity with the slender opening and the closing being capped with the helix, α6. The hydrophobic binding pocket of McinOBP4 was formed by the residues of leucine phenylalanine, tryptophan, valine, and methionine).

## Discussion

Insects OBPs may act as a key role player to transporting odorant molecules from the sensilla lymph to the chemo-receptors, which subsequently translate queues into physiological signals that initiate behavioral responses. Due to the sensitivity and selectivity of OBPs to different odorants, its functions considers as a key point for developing and select an effective control tactics for used in ecologically-friendly pest management strategies which offer alternatives to chemical based control methods (Qiao et al., 2009; He et al., 2011; Ahmed et al., 2014; Yin et al., 2015; Sun et al., 2016). McinOBP4 has conservative 6 cysteines which consistent with published report (Zhou, 2010). Quantitative examination at transcript levels show that McinOBP4 is expressed in male and female antennae but there is very low expression in others tissues (Figure 2). These types' expression phenomena were observed in several OBPs (Zhang et al., 2009; Gu et al., 2010, 2011; Ahmed et al., 2014). Our results indicate that McinOBP4 plays a major role in the olfactory systems response to odorants.

Interaction between OBP protein and odorant ligands were studies in order to know the function and binding mechanisms. Odorant analogs of chemicals released from corn plants damaged by feeding by the Asian corn borer (ACB), *O. furnacalis* (Huang et al., 2009) were selected as ligands in this study. Total 22 corn odorants were selected to test, among them seven, nine, and six compounds are belonging to aldehydes, terpenoids and aliphatic alcohols/aromatic, respectively. Among the tested compounds, limonene (4.12 ± 0.70 μM), trans-3-hexen-1-ol-acetate (6.99 ± 1.23 μM), linalool (9.61 ± 0.23 μM), trans-2-octanal (11.78 ± 0.57), and β-ionone (11.92 ± 0.49 μM) have the highest binding affinities to McinOBP4-Wt at pH 7.4. McinOBP4 not only binds corn green leaf volatiles, including aldehydes and terpenoids, but also binds aliphatic alcohols, in the order limonene>trans-3-hexen-1-ol-acetate>linalool>trans-2-octanal>β-ionone (Figures 3B–D). These compounds are known to attractants to ACB (Huang et al., 2009), the meadow moth, *Loxostege sticticalis* L. (Yin et al., 2012), and the Braconidae wasp *Microplitis mediator* Haliday (Yu et al., 2010). This suggests that McinOBP4 act as a general OBP in *M. cingulum* and which recognize general odorants compounds from undamaged, and mechanically injured, or herbivore damaged corn plants to used host localizations.

Binding affinity of insect OBP is influenced by pH, which as investigated for 1-NPN to McinOBP4-Wt and McinOBP-M1 over a pH range from 4.4 to 9.4. In this pH ranges, the C-terminal eight residues had no effect on the protein functional activity. These results were consisted with the HarmOBP7 in *Helicoverpa armigera* (Sun et al., 2013). In pH 4–5, BmorPBP1 changed their structure through the forming additional α-helix (7th) (Horst et al., 2001; Damberger et al., 2007), but did not occur in the McinOBP4 protein. The pH-dependent ligand release mechanism has been reported in *Culex quinquefasciatus*, CquiOBP1 (Mao et al., 2010), *Aedes aegypti*, AaegOBP1 (Leite et al., 2009) and *Anopheles gambiae*, AgamOBP1 (Wogulis et al., 2006). The binding affinity of the corn odorants to the mutant McinOBP4-M1, showed weak binding affinities with respect to the wild type, unlike to 1-NPN. This suggested that C-terminus might, act as a lid on the binding cavity, and may help to stabilize the odorant complex, but have no involvement in 1-NPN binding.

The second mutant M2 of McinOBP4 has a binding affinity to corn odorants limonene, trans-3-hexen-1-ol-acetate, linalool, trans-2-octanal, and β-ionone that was much weaker compared to the wild type and first mutant (M1, Figure 4C). This result suggests Met119 might be involved in the binding to terpenoids as well as aldehyde and alcohol odorants through the hydrophobic chain in the cavity and the functional group on the mount of the cavity. Previous reports also are in agreement with our present results (Vincent et al., 2000; Spinelli et al., 2012; Sun et al., 2013; Ahmed et al., 2014), that often different ligands might bind equally well to the same OBP protein. Presumably, different orientations in the binding pocket, and different residues might make it possible to residues participate in the protein-ligand interactions.

Comparing our results with other OBPs, we concluded that hydrogen bond and hydrophobic interaction play key roles in the ligand binding specificity of McinOBP4 and provide a reliable and detailed olfactory map of odorant-protein interaction. The C-terminus Met119 may appear involved in binding with limonene, trans-3-hexen-1-ol-acetate as corn odorants. Taken together of present findings suggest that, McinOBP4 could be involved in the recognition of corn odorants, and may serve as a target for insect pest management strategies against ACB that are more environmentally-friendly compared to chemical insecticides.

## Author contributions

The experimental plan conceived and designed by: TA, TZ, and ZW. The experiments performed by: TA. The data processed and analyzed by: TA, TZ, and SB. Writing and editing manuscripts: TA, ZW, TZ, and KH.

### Conflict of interest statement

The authors declare that the research was conducted in the absence of any commercial or financial relationships that could be construed as a potential conflict of interest.
